# Stearns’ Dose Book

**Published:** 1893-08

**Authors:** 


STEARNS' DOSE BOOK. Compiled especially for the use of Physicians and Pharmacists. Presented with the compliments of Frederick Stearns & Co., Manufacturing Pharmacists, Detroit, Michigan.

This little pamphlet of 32 pages contains a variety of matter useful to the physician, such as the doses in the regular school of all remedies, a table of antidotes to poisons, directions for examination of urine, list of disinfectants, and a list of new remedies.

